# Comparing Brain Functional Activities in Patients With Blepharospasm and Dry Eye Disease Measured With Resting-State fMRI

**DOI:** 10.3389/fneur.2021.607476

**Published:** 2021-10-27

**Authors:** Changqiang Feng, Wenyan Jiang, Yousheng Xiao, Yang Liu, Lulu Pang, Meilan Liang, Jingqun Tang, Yulin Lu, Jing Wei, Wenmei Li, Yiwu Lei, Wenbin Guo, Shuguang Luo

**Affiliations:** ^1^Department of Neurology, The First Affiliated Hospital of Guangxi Medical University, Nanning, China; ^2^Department of Comprehensive Internal Medicine, Guangxi Medical University Affiliated Tumor Hospital, Nanning, China; ^3^Department of Radiology, The First Affiliated Hospital of Guangxi Medical University, Nanning, China; ^4^Department of Psychiatry, National Clinical Research Center for Mental Disorders, The Second Xiangya Hospital of Central South University, Changsha, China

**Keywords:** blepharospasm, dry eye disease, functional magnetic resonance imaging, amplitude of low-frequency fluctuations, sensorimotor integration

## Abstract

**Background:** Blepharospasm (BSP) and dry eye disease (DED) are clinically common diseases characterized by an increased blinking rate. A sustained eyelid muscle activity may alter the cortical sensorimotor concordance and lead to secondary functional changes. This study aimed to explore the central mechanism of BSP by assessing brain functional differences between the two groups and comparing them with healthy controls.

**Methods:** In this study, 25 patients with BSP, 22 patients with DED, and 23 healthy controls underwent resting-state functional magnetic resonance imaging (fMRI) scan. The amplitude of low-frequency fluctuations (ALFF) was applied to analyze the imaging data.

**Results:** Analysis of covariance (ANCOVA) revealed widespread differences in ALFF across the three groups. In comparison with healthy controls, patients with BSP showed abnormal ALFF in the sensorimotor integration related-brain regions, including the bilateral supplementary motor area (SMA), left cerebellar Crus I, left fusiform gyrus, bilateral superior medial prefrontal cortex (MPFC), and right superior frontal gyrus (SFG). In comparison with patients with DED, patients with BSP exhibited a significantly increased ALFF in the left cerebellar Crus I and left SMA. ALFF in the left fusiform gyrus/cerebellar Crus I was positively correlated with symptomatic severity of BSP.

**Conclusions:** Our results reveal that the distinctive changes in the brain function in patients with BSP are different from those in patients with DED and healthy controls. The results further emphasize the primary role of sensorimotor integration in the pathophysiology of BSP.

## Introduction

Characterized by excessive involuntary spasms of the orbicularis oculi, blepharospasm (BSP) is now recognized as a common form of adult-onset focal dystonia (1, 2). As disease progresses, it may result in difficulty in opening the eyes or even functional blindness, which causes functional disability in work and daily life and decreases quality of life (3–5). Though the symptomatology of this disease is well-defined, its pathophysiology remains unclear. In addition, the onset of BSP is insidious and mainly characterized by atypical symptoms such as increasing blinking and photophobia in early stages (6). These early symptoms of patients with BSP are very similar to the manifestations of dry eye disease (DED), which is considered a peripheral disease associated with tear hyperosmolarity and ocular surface inflammation (7). Typical manifestations of DED include photophobia, burning sensation, dryness of the eyes, and visual impairment. These unpleasant sensations in DED often initiated secondary increased blink rate. This might be a result of increased reflex non-spasmodic closure of the eyelids to the sensory symptoms other than the involuntary orbicularis oculi spasms in BSP. It is usually difficult to differentiate BSP in early stages from DED merely according to clinical symptoms. Differences in neural activity between the two diseases remain unclear, and sustained muscular activity might alter the cortical sensory-motor concordance directly or indirectly, leading to secondary structural or functional changes that are almost indistinguishable from the underlying pathophysiological characteristics of BSP. It is necessary to identify the potential central effects of peripheral sensory feedback.

Although BSP was initially believed to be a disease of the basal ganglia, neuroimaging evidence suggested that it involved the anatomy and function of several brain regions (8, 9). Promising neuroimaging techniques have provided new methods for analyzing changes in brain function and structure, thereby expanding the focus of traditional research. Structurally, voxel-based morphometry analysis has shown an increased gray matter density in the cingulate gyrus, primary sensorimotor cortex, and right middle frontal gyrus, indicating that multiple regions contribute to the development of BSP (10, 11). Functionally, many fMRI trials on BSP have revealed abnormal regional spontaneous brain activities and altered functional connectivity in sensorimotor structures, including the basal ganglia, SMA, cerebellum, primary sensorimotor cortex, and visual areas (12–16). These studies have demonstrated that local microstructural and functional abnormalities in multiple regions may be associated with BSP, thus bringing forward the viewpoint that BSP might be a network disorder (17). However, these functional and structural changes in BSP are unable to definitely divide into primary or secondary. Whether imaging abnormalities in these areas are characteristic spontaneous changes in BSP or secondary central effects caused by an increased blinking rate should be further investigated. Moreover, little is known about brain activity alterations in DED with commonly recognized peripheral mechanisms. Both disease situations present with similar clinical manifestations, especially increased blinking. To improve the understanding of spasmodic conditions, specific functional changes of BSP should be distinguished from possible confounding effects through dialectical comparative analysis. Therefore, DED may be used as controls to investigate the neural mechanisms of BSP.

In early studies about BSP, an activation paradigm evoked by simple motor tasks was used. However, different abnormalities have shown in the task-based fMRI studies during different task performance (18–20). The lack of a unified stimulus paradigm may make it difficult to support each other across the existent task-based studies. Besides, individual differences may activate the unrelated brain regions during performing the motor task. In contrast to task-based fMRI, increasing attention has been paid to resting-state fMRI (rs-fMRI), a promising neuroimaging technique that can examine brain neurophysiological processes without the requirement of specific task execution based on the fluctuation of the blood-oxygen-level dependent signal. The technique can avoid the potential limitations of task-based fMRI and contributes to further understanding of abnormalities of neural activity. As an effective analytical method of rs-fMRI technique, amplitude of low-frequency fluctuations (ALFF) is widely utilized to identify functional changes in brain regions at rest (21–24). In the present study, we used the ALFF method to explore the different types of alterations in the spontaneous regional brain activity among patients with BSP, patients with DED, and healthy controls. We also examined correlations between abnormal ALFF and clinical variables in patients with BSP. We hypothesized that ALFF alterations in BSP would be distinguishable from those in patients with DED and healthy subjects.

## Methods and Materials

### Participants

All participants in the present study volunteered to take part in the study and signed a written consent after they were informed of the research process. The study received ethics approval by the Ethics Committee of the First Affiliated Hospital of Guangxi Medical University. Patients with BSP and DED were treated at the Department of Neurology in the First Affiliated Hospital of Guangxi Medical University from October 2017 to October 2019.

#### Patients With BSP

Inclusion criteria: (1) blepharospasm in accordance with clinical diagnostic criteria (25); (2) no organic changes in conventional head MRI; (3) no other serious neurological or mental diseases; (4) not received botulinum toxin treatment or any medication for dystonia or mental illness within 3 months before enrollment; and (5) right-handedness. Exclusion criteria: (1) abnormal intracranial lesions found *via* MRI; (2) dry eye, eyelid aphasia, or severe systemic diseases, such as hepatolenticular degeneration; (3) previous history of neurological and mental diseases; and (4) inability to cooperate with or contraindicate MRI examination.

#### Patients With DED

Inclusion criteria: (1) conform to the diagnostic criteria in accordance with the *TFOS DEWS II Definition and Classification Report* (26); (2) no accompany blepharospasm; (3) not received botulinum toxin, and without any drugs for dystonia or mental illness within 3 months before enrollment; and (4) right-handedness. Exclusion criteria: (1) orbital organic lesions confirmed by orbital CT examination; (2) head and facial trauma or operation history and neuropsychiatric system diseases; (3) abnormal intracranial lesions found through imaging examination; and (4) inability to cooperate with or unsuitable for MRI examination.

#### Healthy Controls

Healthy adults from the local community were recruited as healthy controls and matched with age and education with subjects with BSP and patients with DED. Inclusion criteria: (1) no neurological and mental system diseases; (2) no abnormality in ophthalmology and nervous system examination; (3) no abnormality in head imaging examination; and (4) right-handedness.

### Resting-State fMRI Images Acquisition

Images were collected by the same skilled technician using a Siemens 3T MR scanner in the First Affiliated Hospital of Guangxi Medical University. All subjects were given head retainers and earplugs to minimize head movement and machine noise. During scanning, all participants were required to relax and minimize head and body movement as much as possible, while staying awake with eyes closed. An echo-planar imaging sequence was used to acquire resting-state fMRI images with the following parameters: repetition time/echo time = 2,000 ms/30 ms, matrix size = 64 × 64, slices number = 30, flip angle = 90°, field of view = 24 cm, gap = 0.4 mm, slice thickness = 4 mm, and 250 volumes (500 s).

Clinical information, including age, gender, illness duration (except healthy control groups), education level, and BSP symptomatic severity, were collected. The symptom severity of patients with BSP was assessed by professional neurologists utilizing the Jankovic Rating Scale (JRS) (27). In addition, patients with BSP were assessed in terms of their depression and anxiety symptoms by using, respectively, the self-rating depression scale (SDS) and self-rating anxiety scale (SAS).

### Data Preprocessing

Data Processing Assistant for Resting-State fMRI (DPARSF) software package based on MatlabR2012b platform was used to perform data preprocessing. First, images of the first 10 time points were eliminated, and the remaining 240 images were retained for analysis. Time layer and head motion were corrected to eliminate data whose head movement horizontal displacement was >2 mm and rotation was >2°. Next, the images were spatially normalized to the Montreal Neurological Institute (MNI) EPI template and resampled to 3 mm × 3 mm × 3 mm in the statistical parameter map software (SPM12, http://www.fil.ion.ucl.ac.uk/spm). Afterward, the processed image was spatially smoothened with a 4 mm full width at half maximum Gaussian kernel. Finally, linear drifts removal and temporal band-pass filtering (0.01–0.08 Hz) were conducted on all smoothed images to remove the influence of low-frequency drift and high-frequency respiratory and cardiac noise.

### ALFF Analysis

The REST (http://resting-fmri.sourceforge.net) software was used to analyze ALFF (28). ALFF is the sum of the low-frequency spectral amplitudes across 0.01–0.08 Hz. First, the time series of each voxel was transformed to the frequency domain by utilizing a fast Fourier transform and the power spectrum was obtained. Then, the square root of each frequency on the power spectrum was calculated, and the mean square root across 0.01–0.08 Hz at each voxel was taken as the ALFF (29). For data standardization, the ALFF of each voxel was divided by the global mean ALFF value.

### Statistical Analysis

Demographic data, such as gender, age, and education level, were statistically analyzed using IBM SPSS 23.0 software. Analyses of variance (ANOVA) were conducted to compare age and education level across the three groups, and gender differences were compared *via* a chi-square test. A two-sample *t*-test was used to compare illness duration between the BSP group and the DED group. The threshold of above tests was set at *p* < 0.05 that indicated statistically significant differences.

ANCOVA was performed to compare the ALFF images across three groups with the REST software. Subsequently, *post-hoc t*-tests were carried out to examine ALFF differences between each pair of groups. Age, gender, and education were used as covariates to reduce their potential impact. The significance threshold was corrected to *p* < 0.05 for multiple comparisons using Gaussian random field (GRF) theory (voxel significance: *p* < 0.001, cluster significance: *p* < 0.05).

The mean ALFF values were extracted from abnormal brain regions showing significant differences in BSP group and DED group by between-group comparisons for further correlation analysis.

Pearson correlation or Spearman correlation analyses were performed to examine the association of ALFF values and clinical variables in patients with BSP and patients with DED when apposite. Significance level was set at *p* < 0.05 (corrected for Bonferroni correction).

## Results

### Demographics and Clinical Information

A total of 70 participants were included in the study: 25 patients with BSP, 22 patients with DED, and 23 healthy controls. Demographics and clinical characteristics of these participants are shown in Table 1. There was no significant difference in age and education level across the three groups. Moreover, we observed that 20 patients of BSP (80.00%) exhibited sensory tricks, a characteristic feature in primary dystonia. That is, stimulus such as touching the cheeks and wearing glasses could temporarily alleviate the symptom of eyelid spasms.

**Table 1:** Demographic information and clinical profile of the participants.

**Variables**	**BSP** **(*n* = 25)**	**DED** **(*n* = 22)**	**Healthy** **controls** **(*n* = 23)**	***p*-value**
Gender (male/female)	8/17	8/14	4/19	0.332^a^
Age (years)	49.68 ± 8.41	51.82 ± 8.68	49.69 ± 6.51	0.583^b^
Education (years)	10.32 ± 2.30	10.23 ± 3.42	9.78 ± 2.59	0.746^b^
Illness duration (months)	11.64 ± 9.01	21.77 ± 17.30		0.019^c^
Symptom severity	2.64 ± 0.81			
SAS	42.52 ± 10.18			
SDA	48.51 ± 8.86			

*BSP, blepharospasm; DED, dry eye disease; SAS, Self-rating anxiety scale; SDS, Self-rating depression scale*.

a *The p-value for sex distribution was obtained by a chi-square test*.

b *The p-values were obtained by analyses of variance*.

c *The P-values were obtained by a two-sample t-test*.

### Group Differences in ALFF

**Figure 1:**
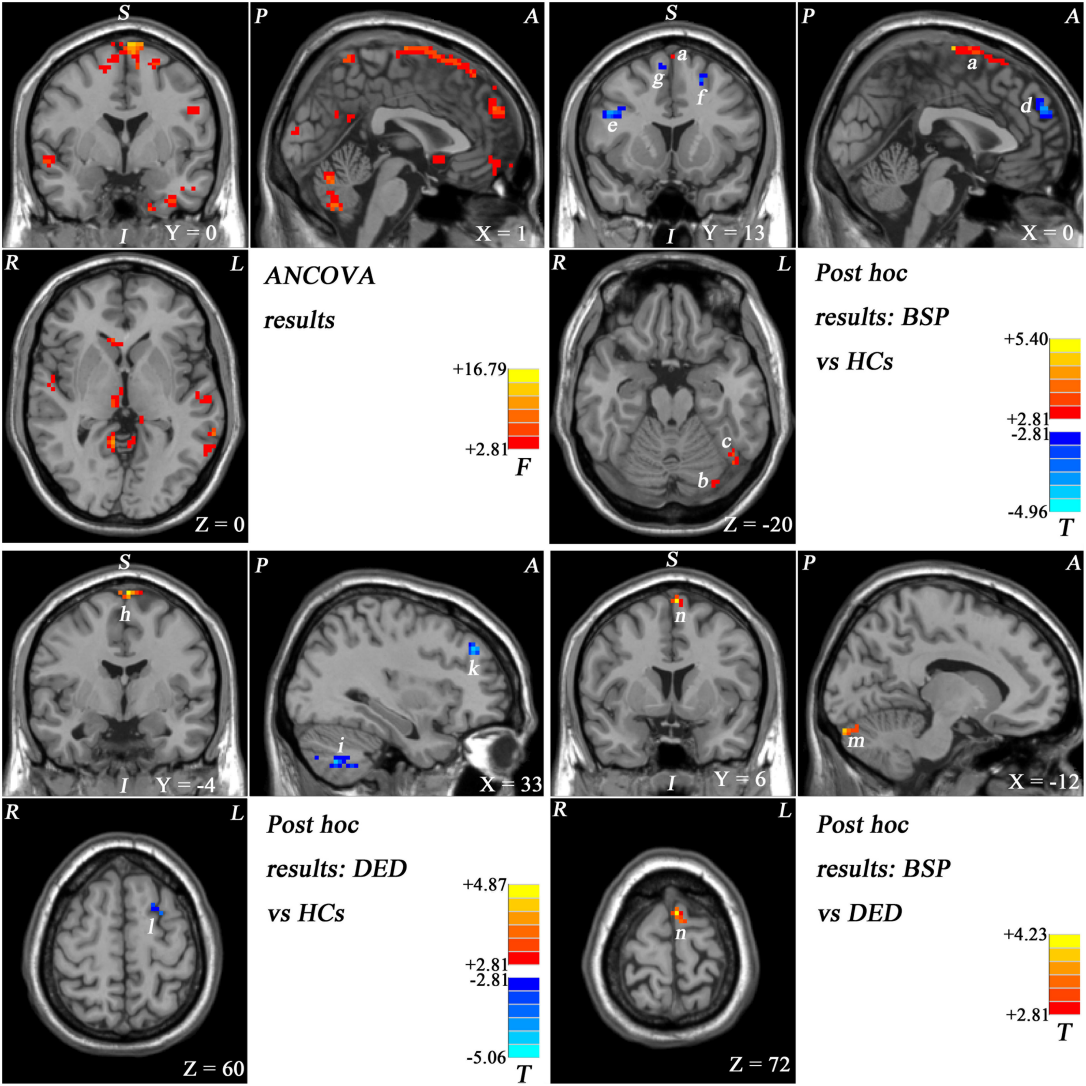
Statistical maps showing ALFF values differences in different brain regions across studied groups. Red and blue represent higher and lower ALFF, respectively. The color bars indicate the *F*-values from the ANOVA analyses and the *T*-values from *post-hoc* analysis between each pair of groups. (a) Bilateral SMA, (b) left cerebellar crus I, (c) left fusiform gyrus/cerebellar crus I, (d) bilateral superior MPFC, (e) right inferior frontal gyrus, (f) left middle frontal gyrus, (g) right superior frontal gyrus, (h) bilateral SMA, (i) right cerebellar VIII/VIIb, (k) right middle frontal gyrus, (l) left middle frontal gyrus, (m) left cerebellar crus I, and (n) left SMA.

As shown in Figure 1 and Supplementary Figure 1, the ALFF values were significantly different in the frontal and cerebellar regions across the three groups by ANCOVA.

Patients with BSP showed increased ALFF in the bilateral SMA, left cerebellar Crus I, and left fusiform gyrus/cerebellar Crus I and decreased ALFF in the superior part of the bilateral MPFC, right SFG, left middle frontal gyrus, and right inferior frontal gyrus compared with healthy controls (Figure 1; Supplementary Figure 2; Table 2).

**Table 2:** Regions with abnormal ALFF in the patients.

**Cluster location**	**Peak (MNI)**			**Number of** **voxels**	***T*-value^**a**^**
	***x***	***y***	***z***		
**BSP vs. controls**					
Bilateral SMA	−3	−12	78	119	5.3996
Left cerebellar crus I	−33	−81	−24	46	4.2125
Left fusiform gyrus/cerebellar crus I	−45	−63	−18	25	5.1248
Bilateral superior MPFC	3	57	30	35	−4.4314
Right inferior frontal gyrus	48	15	27	37	−4.9554
Left middle frontal gyrus	−24	9	51	23	−4.1464
Right superior frontal gyrus	6	18	57	59	−4.8495
**DED vs. controls**					
Bilateral SMA	−3	−3	78	45	4.8692
Right cerebellar VIII/VIIb	18	−69	−51	128	−5.0609
Left cerebellar VIII/VIIb	−18	−72	−42	172	−4.4160
Right middle frontal gyrus	33	39	36	21	−4.6227
Left middle frontal gyrus	−27	6	60	26	−3.5618
**BSP vs. DED**					
Left cerebellar crus I	−12	−93	−24	27	3.9253
Left SMA	−3	6	72	25	4.2264

*MNI, Montreal Neurological Institute; ALFF, amplitude of low-frequency fluctuations; MPFC, medial prefrontal cortex; ACC, anterior cingulate cortex; SMA, supplementary motor area*.

a *A positive/negative T-value represents increased/decreased ALFF*.

Patients with DED exhibited increased ALFF in the bilateral SMA and decreased ALFF in the bilateral cerebellar VIII/VIIb, left middle frontal gyrus, and right middle frontal gyrus compared with that of healthy controls (Figure 1; Supplementary Figure 3; Table 2).

Compared with patients with DED, patients with BSP showed significantly increased ALFF in the left SMA and left cerebellar Crus I (Figure 1; Supplementary Figure 4; Table 2).

### Correlation Analysis

**Figure 2:**
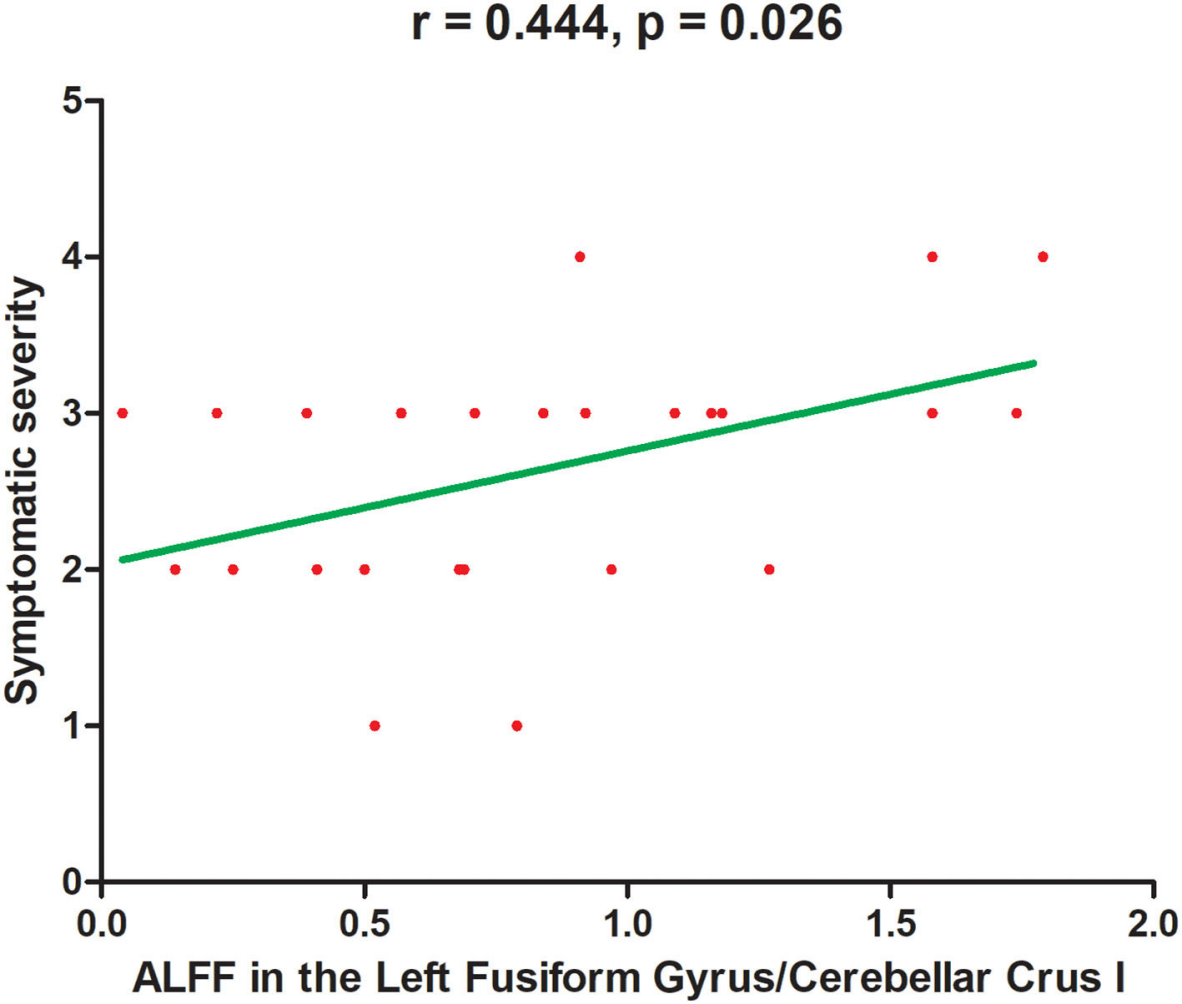
A significantly positive correlation between symptomatic severity and ALFF values in the left fusiform gyrus/cerebellar crus I in patients with BSP. ALFF, amplitude of low-frequency fluctuations. *X*-axis represents ALFF *z*-score. *Y*-axis represents symptomatic severity.

A significantly positive correlation was observed between ALFF values in the left fusiform gyrus/cerebellar Crus I and symptomatic severity (*r* = 0.444, *p* = 0.026) in patients with BSP by using a Spearman correlation analysis (Figure 2). No significant correlation was revealed between abnormal ALFF and other clinical variables of patients with BSP. No correlation between abnormal ALFF and clinical variables was observed in the subjects with DED.

## Discussion

The present study was the first to explore functional changes in brain regions of patients with BSP, patients with DED, and healthy controls by measuring ALFF of fMRI signals at rest. Abnormal ALFF values were located in the sensorimotor integration related brain regions in patients with BSP, including the bilateral SMA, left cerebellar Crus I, left fusiform gyrus, bilateral superior MPFC, and right SFG compared with healthy controls. Patients with BSP exhibited significantly increased ALFF in the left cerebellar Crus I and the left SMA compared with patients with DED. In addition, ALFF in the left fusiform gyrus/cerebellar Crus I was positively correlated with the symptomatic severity of BSP.

Dystonia was previously described as a movement disorder resulting from basal ganglion dysfunction, but this description has been remarkably revised, and the role of sensorimotor integration in the pathophysiology of dystonia has been gradually recognized (30–33). In general, sensorimotor integration refers to all processes that sensory information is used to plan and carry out volitional movements, and the sensory counterparts of each implemented movement (34). Indeed, patients with dystonia exhibit not only motor symptoms but also some sensory field disorders, including abnormalities in sensory and perceptual functions (35–38). For example, patients with BSP often complain to have photophobia and other ocular discomforts, and neck pain is frequently associated with cervical dystonia (39, 40). In addition, a well-known clinical feature of dystonia is the sensory trick that can alleviate dystonic symptoms in some ways (41–44). Similarly, touching certain areas of the face, wearing colored glasses, or singing can relieve the spasm symptom in patients with BSP (45, 46). In our study, 20 patients of BSP (80.00%) presented with sensory tricks, which might be the adjustment of motor output through the modification of sensory input. Sensorimotor integration is a complex neural activity involving multiple brain regions and circuits, most of which are component parts of the sensorimotor network (SMN) (47). This network mainly involves action execution and sensory processing, and the SMA is one of the key nodes (48). The SMA receives signals from the basal ganglia and cerebellum and then delivers them to primary motor cortex to coordinate motion execution instructions and participate in sensorimotor integration (36, 49, 50). Many structural and functional MRI studies on dystonia, including BSP, have usually revealed abnormalities in the SMA, and animal models of dystonia have shown overexcitability and increased proprioceptive input in the SMA (51–54). In our study, high ALFF was observed in the bilateral SMA in patients with BSP and patients with DED, and this result might be caused by some interference from peripheral paresthesia components. However, patients with BSP exhibited increased ALFF in the left SMA compared with patients with DED, indicating that this region might be a primary change in BSP. Speculatively, the present observation of increased ALFF in the left SMA may reflect the possibility that abnormal motor information from SMA to primary motor cortex results in improper motor outflow, creating involuntary closure of the orbicularis oculi that characterizes BSP.

The function of the cerebellum in motion control and coordination has been traditionally accepted, and further research evidence has emphasized the critical role of the cerebellum in the pathophysiology of dystonia (55, 56). For instance, diffusion tensor imaging studies have demonstrated abnormal integrity of cerebellar-thalamo-cortical fiber tracts in isolated focal dystonia phenotypes and decreased fractional anisotropy in the right cerebellum of patients with cervical dystonia (57, 58). Similarly, we found increased ALFF in the left cerebellar Crus I in patients with BSP compared with healthy controls and patients with DED. In addition, no correlation was seen between the increased ALFF in the left cerebellar crus I and JRS scores or symptomatic severity in BSP patients, suggesting that the abnormal ALFF values in the left cerebellar crus I might be independent of the illness duration and disease severity. The cerebellum receives fibers from the brainstem cerebellar pathway and projects them to the sensorimotor cortex through the cerebellar Crus I/II to modulate muscular tone and coordinate muscle groups (59, 60). In addition, separate cortico–cerebellar circuits can engage visual–spatial processing and response to the visual stimulus, supporting the assumption that the cerebellum is also involved in vision modulation (61). There is increased ALFF in the left cerebellar Crus I of patients with BSP, suggesting that the cerebellum might be another key node that received abnormal visual sensation and muscle tension caused by persistent eye spasm. As a consequence, it becomes hyperactive to regulate abnormal movement through sensorimotor integration process. Therefore, alterations in the left cerebellar Crus I might be distinctive in BSP, and an increased cerebellar neuronal activity might induce subsequent adaptive activities in motor systems. Traditional deep brain stimulation (DBS) and TMS therapeutic targets such as the globus pallidus interma and subthalamic nucleus of dystonia have expanded to the cerebellum in certain types of dystonia (62, 63). Cerebellum could be a potential target for in-depth exploration of intervention strategies in BSP.

Interestingly, our results showed an increased ALFF in the left fusiform gyrus and a positive correlation between ALFF in this region and symptomatic severity in patients with BSP. As part of the primary visual network, the fusiform gyrus is an important link in the human ventral visual information processing pathway that processes color information and facial expression perception (64, 65). A wide range of cognitive deficits are found in patients with BSP; among them, visuospatial function is the most frequently affected area (66). The characteristics of this visuospatial function abnormality was mutually supported by our results, which might be distinctive visual network changes secondary to aberrant visual input caused by eyelid spasms. Moreover, visual areas are widely connected to the basal ganglia and other motor areas. Thus, ALFF alterations in the left fusiform gyrus might result in visual-motor integration dysfunction, a form of sensorimotor integration. The positive correlation between ALFF in the left fusiform gyrus and symptomatic severity of BSP might represent an adaptive role or reaction to abnormal visual information.

We also found decreased ALFF in the bilateral superior MPFC and right SFG in patients with BSP. The MPFC and SFG are core components of the brain's default mode network (DMN), which is commonly recognized to be involved in cognitive functions and emotional processes (67). Our previous study in patients with BSP revealed an enhanced homotopic coordination in the inferior temporal gyrus and posterior cingulate cortex within the DMN (15). These abnormalities indicated distinct differences within the DMN in patients with BSP. One possibility is that these changes within the DMN might be related to nonmotor symptoms, such as anxiety, depression, and cognitive impairments, in patients with BSP; and these changes occur more frequently in patients with BSP than that in healthy controls (9). Although our results showed no significant difference in SAS/SDS scores between patients with BSP and healthy controls, it still raises a possibility that our patients might have subclinical emotional and cognitive impairments. A widely accepted viewpoint proposed that the MPFC is a high-level nervous center located at the top of the information processing stream, which flexibly map sensory signals to motor actions and achieve an adaptive choice behavior (68–70). It can be seen that the MPFC plays a role in sensorimotor integration. Moreover, MPFC has been thought to involve higher-level cognitive functions related to voluntary action, including formation of movement plans and choice of executive actions (71, 72). Likewise, the SFG is connected to the critical nodes of the motor-related network, including the precentral gyrus, caudate nucleus and thalamus (73). In this regard, the DMN might partly contribute to motor regulation apart from non-motor symptoms in BSP through functional connectivity.

In addition to small sample size, some limitations should be noted in this study. First, the potential dystonic activity of the eyelids during fMRI scanning should be considered although all subjects denied this phenomenon. Second, we were unable to detect the potential dysfunction of connections across different brain regions of patients with BSP because the ALFF measurements do not reflect the integration of the brain function activity at a network level. Furthermore, it should be noted that the application of head retainers and earplugs during images acquisition might induce sensory tricks, thus disturbing the “resting-state” to some extent.

## Conclusion

Despite the limitations, the design of combining patients with BSP and patients with DED may be a feasible option to distinguish the secondary central effects of the sustained muscle activity in neuroimaging studies on BSP. In conclusion, our results reveal that the distinctive changes in the brain function of patients with BSP are different from those in patients with DED and healthy controls. The results further emphasize the primary role of sensorimotor integration in the pathophysiology of BSP.

## Data Availability Statement

The raw data supporting the conclusions of this article will be made available by the authors, without undue reservation.

## Ethics Statement

The studies involving human participants were reviewed and approved by the Ethics Committee of the First Affiliated Hospital of Guangxi Medical University. The patients/participants provided their written informed consent to participate in this study.

## Author Contributions

SL, WG, CF, and WJ made substantial contributions to the conception of the work. YiL and WL did the acquisition of data. YX, YaL, YuL, JT, JW, LP, and ML analyzed the data. CF and WJ wrote the main manuscript text. All authors reviewed and approved the manuscript.

## Funding

This study was supported by grants from the National Natural Science Foundation of China (Grant No. 81771447), National Key R&D Program of China (Grant No. 2016YFC1307100), Guangxi Appropriate Technology for Medical and Health Research Development Project (Grant No. S2020028), and Incubation Project of Research Team (Grant No. MINKEFY202108).

## Conflict of Interest

The authors declare that the research was conducted in the absence of any commercial or financial relationships that could be construed as a potential conflict of interest.

## Publisher's Note

All claims expressed in this article are solely those of the authors and do not necessarily represent those of their affiliated organizations, or those of the publisher, the editors and the reviewers. Any product that may be evaluated in this article, or claim that may be made by its manufacturer, is not guaranteed or endorsed by the publisher.
