# Supplementary material for: Comparing Brain Functional Activities in Patients With Blepharospasm and Dry Eye Disease Measured With Resting-State fMRI
Source: Front Neurol. 2021 Oct 27;12:607476. doi: 10.3389/fneur.2021.607476 (PMC8578056; doi:10.3389/fneur.2021.607476)
Supplement: Supplementary file 1 [file Presentation_1.pdf]

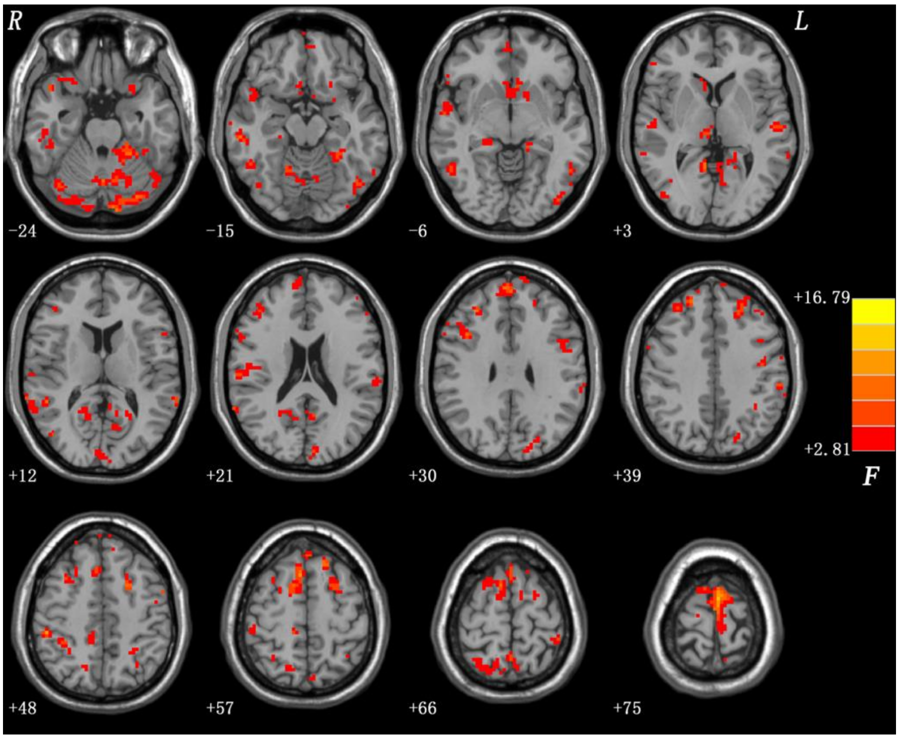

Figure S1. ANCOVA results showed the ALFF values differences in different brain regions across BSP, DED groups and healthy controls. Red denotes increased ALFF. The color bars denote the F values from the ANOVA analyses. ANCOVA=analyses of covariance; ALFF=amplitude of low-frequency fluctuations; BSP=blepharospasm; DED=dry eye disease.

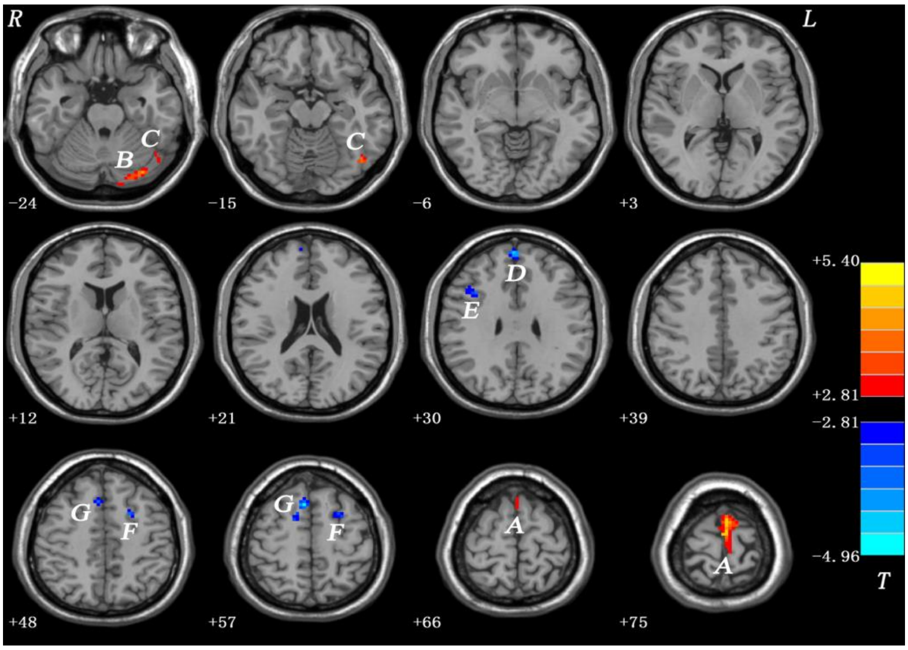

Figure S2. Brain regions showing significant ALFF differences between patients with BSP and healthy controls. Red and blue represent higher and lower ALFF respectively.

The color bars denote the T values from post hoc analysis between BSP group and healthy controls group. Brain regions were labeled as follows: A: bilateral SMA; B: left cerebellar Crus I; C: left fusiform gyrus/cerebellar Crus I; D: bilateral superior MPFC; E: right inferior frontal Gyrus; F: left middle frontal Gyrus; and G: right superior frontal gyrus.

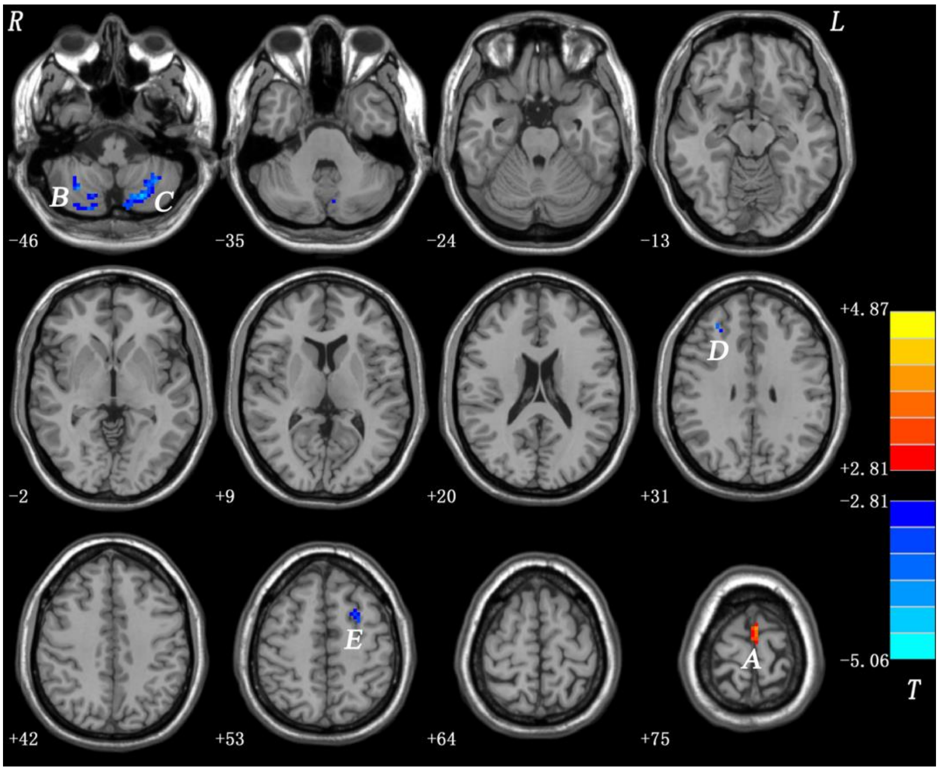

Figure S3. Brain regions showing significant ALFF differences between patients with DED and healthy controls. Red and blue represent higher and lower ALFF respectively. The color bars denote the T values from post hoc analysis between DED group and healthy controls group. Brain regions were labeled as follows: A: bilateral SMA; B: right cerebellar VIII/VIIb; C: left cerebellar VIII/VIIb; D: right middle frontal gyrus; and E: left middle frontal gyrus.

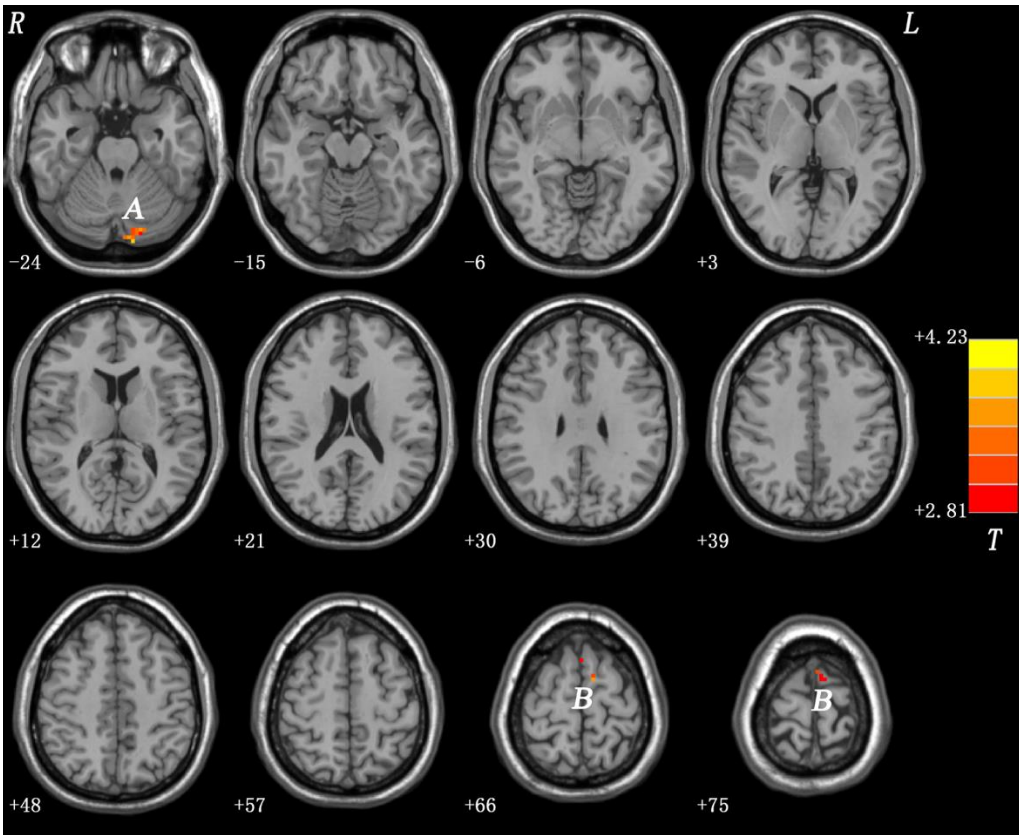

Figure S4. Brain regions showing significant ALFF differences between patients with BSP and patients with DED. Red denotes higher ALFF values. The color bars indicate the T values from post hoc analysis between BSP group and DED group. Brain regions were labeled as follows: A: left cerebellar Crus I; B: left SMA.
